# Micronutrient Biomarkers and Their Association with Malaria Infection in Children in Buea Health District, Cameroon

**DOI:** 10.3390/tropicalmed9120303

**Published:** 2024-12-10

**Authors:** Jerome Nyhalah Dinga, Emmanuel Fondungallah Anu, Romelle Dibanda Feumba, Haowen Qin, Flora Ayah, Rene Bilingwe Ayiseh, Robert Adamu Shey, Stanley Dobgima Gamua, Anthony Kukwah Tufon, Rameshbabu Manyam, Vincent P. K. Titanji

**Affiliations:** 1 Michael Gahnyam Gbeugvat Foundation, Buea, Cameroon; 2 Biotechnology Unit, University of Buea, Buea, Cameroon; 3 Rollins School of Public Health, Emory University, Atlanta, GA 30322, USA; 4 African Vaccinology Network, Buea, Cameroon; 5 Department of Biochemistry and Molecular Biology, University of Buea, Buea, Cameroon; 6 Buea Regional Hospital, Buea, Cameroon; 7 Department of Microbiology and Parasitology, University of Buea, Buea, Cameroon

**Keywords:** micronutrient biomarkers, malaria infection, ferritin, soluble transferrin receptor, retinol-binding protein 4, thyroglobulin

## Abstract

Recently malaria and micronutrient deficiencies have become a major worldwide public health problem, particularly in Africa and other endemic countries with children under 5 years old being the most vulnerable. Apart from nutritional problems that cause micronutrient deficiencies, studies have also reported that parasitic infections like malaria can affect the levels of micronutrients. Thus, this research was aimed at assessing the serum levels of micronutrient biomarkers and their association with malaria infection in children under 5 years old in the Buea Health District. Method: This cross-sectional study recruited 80 participants from February to April 2024. The micronutrient biomarkers levels were measured using a Q-7plex Human Micronutrient Measurement Kit. Results: There were changes in serum micronutrient biomarkers levels between malaria infected and healthy children. Ferritin was higher in sick children (23.53 μg/L ± 7.75) than in healthy children (19.07 μg/L ± 3.87), significantly (*p* < 0.002). The same trend was observed with the soluble transferrin receptor being higher (*p* < 0.049) in sick children (3.74 mg/L ± 1.92) compared to healthy ones (3.08 mg/L ± 0.64). In addition, the levels of retinol-binding protein 4 and thyroglobulin levels were not significantly different between the sick and healthy children. Therefore, this study revealed that malaria causes alterations in the serum levels of micronutrient biomarkers and consequently affects micronutrient levels in children below the age of 5 in the Buea Health District.

## 1. Introduction

Malaria is a parasitic disease caused by *Plasmodia* spp. transmitted to humans through the bite of an infected female *anopheles* mosquito. This study focused on *Plasmodium falciparum*, the most prevalent and pernicious species in the Cameroonian rainforest [1]. In recent decades, malaria has become a major worldwide public health problem, particularly in Africa and other endemic countries [2]. The human malaria parasite has an extensive life cycle that involves two hosts: a human and a mosquito. The parasite is transmitted through a mosquito bite. The released Plasmodium sporozoite invades the liver and later emerges as merozoites that carry out a repeated life cycle in the red blood cells (RBCs). After this repeated cycle, some merozoites differentiate into sex gametes that are taken up by a mosquito during a blood meal to complete the rest of the life cycle [3,4].

The WHO estimated that there were 249 million malaria cases and 608,000 malaria deaths globally in 2022. *Plasmodium falciparum*, out of the five species of *plasmodium*, is the most common malaria parasite in Sub-Saharan Africa, responsible for 95% of estimated malaria cases in 2022. However, about 11,000 people die from malaria in Cameroon every year [2]. The most vulnerable groups to malaria infection are pregnant women and children under the age of five. Meanwhile, micronutrient deficiencies have been implicated as a major public health burden in low- and middle-income countries (LMICs), affecting most especially women of reproductive age and children below 5 years old [5]. These micronutrients include vitamin A, zinc, iron, and iodine and their deficiencies were frequently linked to a higher vulnerability to diseases like malaria [6]. Micronutrient deficiency has been linked to a variety of infections, including malaria-related morbidities. For example, *Plasmodium falciparum* competes for copper, zinc, and iron in its host to multiply and survive [7,8], thus leading to other negative effects on host’s immunity. Therefore, micronutrient surveillance is necessary for providing an early warning of a risk of deficiency [9]. Micronutrient levels have been assessed by measuring their biomarkers. Some of these biomarkers include the retinol binding protein 4 (RBP4) for vitamin A in the form of retinol, thyroglobulin (Tg) for iodine, soluble transferrin receptors (sTfR) for functional iron levels, and ferritin for the storage of iron [10].

**Figure 1:**
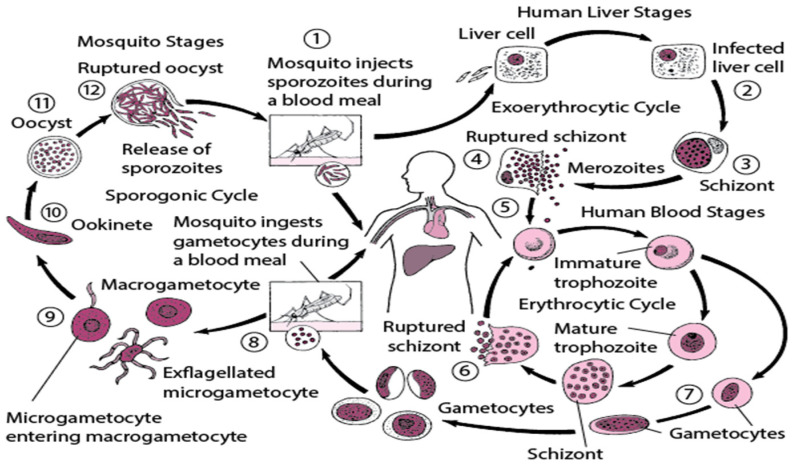
Shows the lifecycle of a malaria parasite.

The malaria parasite life cycle occurs in two different hosts (mosquito and human hosts). Figure 1 [11] below is a detailed diagrammatic representation of the lifecycle of the malaria parasite.

RBP4 is a vital transporter that transports vitamin A in the form of retinol, from the liver to the peripheral tissues [12]. Studies show that vitamin A in the form of retinol is used for immune cell development [13]. RBP4, whose primary role is to transport retinol from the liver to the other tissues, is also produced in the liver. Its production is dependent on the available functional vitamin A in the liver; thus, if they are low, it means the levels of the transport protein will be low, showing a deficiency in vitamin A levels. Vitamin A is required for the development and function of T cells, a type of white blood cell that plays an important role in the immune response to malaria and is also important for vision and bone development in children [14]. Vitamin A deficiency can impair the T-cells’ ability to kill malaria parasites [15].

Ferritin is a protein produced by the liver whose function is to store iron by binding it, and this iron can be used in the production of RBCs and lymphocytes like the B cells of the immune system. In parasitic infections, ferritin releases iron, which is transported through the blood stream to the bone marrow by transferrin to produce immune cells against the infection. Thus, low levels of ferritin indicate that there is low iron storage thus iron deficiency [16]. On the other hand, sTfR are used as functional iron biomarkers, which are located on the surfaces of cells and their main function is to bind to the transferrin protein, which transports iron, thus facilitating iron uptake by the cells. During iron demand by the cells, transferrin receptors are produced, thus releasing a fragments known as sTfR. When there is high iron demand by the cells, more transferrin receptors are produced and subsequently more sTfR fragments are released [10]. High levels of sTfR in the blood means increased cellular iron needs and above normal levels will mean there is functional iron deficiency. During malaria infection, parasites consume hemoglobin within RBCs, leading to iron deficiency and a need for replenishment [17]; thus, more iron is transported to the bone marrow to produce more RBCs [18].

Thyroglobulin is a protein produced by the thyroid gland that serves as a precursor to produce the thyroid hormones [19]. It has been used as a biomarker for iodine [10]. The synthesis of thyroglobulin requires iodine, and low levels of iodine will lead to low production of the thyroid hormone [20]. Thus, to maintain adequate hormone levels, the thyroid gland increases thyroglobulin production in response to low iodine [21]. In addition, studies have shown that malaria infection affects the thyroid gland, by suppressing thyroid hormone production and subsequently reducing thyroglobulin levels [22].

The aim of this study was to assess the serum levels of micronutrient biomarkers in malaria infections in children 0 to 5 years old in the Buea Health District and to assess whether the levels of these biomarkers correlate and are associated with either malaria infection or the absence of it.

## 2. Materials and Methods

### 2.1. Study Design

This was a cross-sectional study, carried out in the Buea Health District, Southwest Region of Cameroon, involving malaria sick and non-malaria sick children within the age of 0 to 5 years.

### 2.2. Study Site

This study was conducted in Buea, Southwest Region of Cameroon. The climate of Buea is of the equatorial type with temperatures that range from 25 to 29 °C annually. It is situated at the foot of Mount Cameroon. There are two main seasons; the rainy season, which starts from June to around October, and the dry season, which starts from November to May. Malaria is endemic in this region and transmission occurs all year round with an average of 1–100 malaria cases per thousand per year [23]. 

### 2.3. Study Population

The study population included children, both male and female subjects within the age range of 0–5 years, who were either diagnosed with malaria or were healthy controls. After a brief explanation about the research objectives, the parents of the children gave their consent. Malaria was diagnosed according to the 2020 guidelines from the World Health Organization (WHO), which include either microscopy or Rapid Diagnostic Test (RDT) techniques. In this study, we used the microscopy technique for the diagnosis, which involves staining and examining a thick blood smear under a microscope to identify malaria parasites. Here, a drop of blood from the patient was dropped on a slide which was used to prepare a thick blood smear and after drying the slide, a Giemsa stain was used to stain the slides for a period of at least 7 to 10 min. The slides were then dried and observed under a microscope using an immersion oil.

### 2.4. Inclusion Criteria

Those included in the present study were:

✔ Children diagnosed with and without malaria.
✔ Children whose parents agreed that their child could take part in the study.
✔ Children aged between 0 and 5 years.

### 2.5. Exclusion Criteria

Those excluded from this study were:

✔ Children with severe health issues.
✔ Children on malaria medication.

The children admitted into the study were not tested for any infection or co-infection.

### 2.6. Sample Collection

Blood samples of 4 mL were collected from each study participants after receiving parental consent for each child through vein puncture. Malaria diagnosis was carried out by microscopy with 43 diagnosed with malaria and 37 healthy children. The blood samples were collected into dry tubes and centrifuged at 3000× *g*. The serum was transferred into Eppendorf tubes and stored at −20 °C, until thawed and used for measuring micronutrient biomarkers at the Biotech Unit, University of Buea.

### 2.7. Measurement of Micronutrient Biomarkers

The 7-Plex Human Micronutrient Measurement Kit was used to measure micronutrient biomarkers per the manufacturer’s instructions [24]. Briefly, prior to use, all the kit components were brought to room temperature. The wash buffer was prepared by diluting 20× 50 mL of wash buffer in 950 mL of distilled water. The 1× sample diluent was prepared by diluting the 2× sample diluent in 10 mL of distilled water. Then, 1 mL of the 1× sample diluent was added to the lyophilized competitor, allowed to sit for 5 min, mixed, and then transferred back into the 19 mL of the 1× sample diluent and mixed thoroughly. This solution was known as the complete Sample diluent. The calibrators were prepared by reconstructing the lyophilized calibrators with the complete sample diluent with volumes that were specified on the certificate that came with the kit. The volumes were mixed and allowed to sit for 5 min. The chemiluminescent substrate was prepared by combining 3 mL of substrate A and 3 mL of the clear substrate B.

### 2.8. Assay Procedure

An 8-point calibration was prepared (7 points and 1 blank) using polypropylene 96 well plate. Then, 200 µL of the prepared calibrator was transferred into the first well and 120 µL of the complete sample diluent was transferred into the 7 wells. Then, 60 µL of the undiluted prepared calibrator was transferred from the first tube into the second tube, mixed thoroughly and the transfer was repeated from tube to tube for 5 more tubes except for the 8th tube that served as the blank. A 1:40 serum sample dilution was prepared by adding 5 µL of the serum sample in 195 µL of sample diluent and mixed thoroughly. The calibrator was run in duplicates with each well taking 50 µL of the calibrator. The same way, samples were run in duplicates by adding 50 µL of each diluted sample into each well. The plates were covered with a plate seal and incubated for 2 h at room temperature. The wells were washed 3 times using 100 µL per well of the wash buffer and the detection mix was added to the plates: 50 µL per well, incubated for 1 h at room temperature. The wells were washed 3 times using 100 µL per well of the wash buffer. Then, 50 µL of Streptavidin-HRP was added per well, then covered using a plate seal and incubated for 20 min at room temperature. The wells were washed 6 times using 100 µL of the wash buffer per well. Then, 50 µL of the chemiluminescent substrate was added per well and imaged immediately using a third-party imager, the Gel Doc imager. The images of the plates were taken and analyzed using the Q-View software. (Quansys Biosciences, Logan, UT, USA) The software compared the dot intensities of the analytes in the samples to that of the calibrated ranges and the software provided the concentrations of the analytes present in each sample.

### 2.9. Data Analysis

Data analysis was performed using Microsoft Excel, SPSS software (version 17.0, Chicago, IL, USA), and R programming language (version 4.2.1). Odds ratios and the effects of RBP4, Ferritin, sTfR, and Tg levels on malaria infections were calculated using logistic regression models in the Python programming language (version 3.10.11). A value of *p* < 0.05 was considered significant. Correlation heatmaps were generated using the Seaborn library in Python.

### 2.10. Ethical and Administrative Approval

Ethical clearance for this study was obtained from the Faculty of Health Science Institutional Review Board (FHSIRB), University of Buea, ref:2023/2171-10/UB/SG/IRB/FHS of 27 November 2023. The objectives of the study were explained to the caregiver of the child after which they voluntarily signed the issued informed consent form.

## 3. Results

### 3.1. Sociodemographic Characteristics of Study Participants

**Figure 2:**
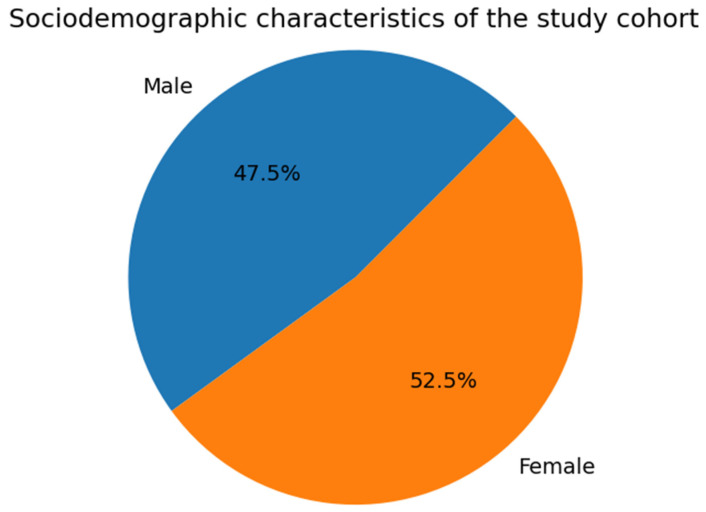
Sociodemographic characteristics of the study cohort.

A total of 80 participants were analyzed in this study for technical reasons. Forty-three participants were diagnosed with malaria. The numerical values of parasite load were: 80 T/µL (n = 14), 120 T/µL (n = 8), 160 T/µL (n = 10), 200 T/µL (n = 10), and 240 T/µL (n = 1). While 37 were healthy children characterized with no fever, diagnosed negative for malaria and any other severe disease (through verbal interview of participant’s parents), having the mean age of 18.3 (months) and a standard deviation of 17.1. Figure 2 shows the sociodemographic characteristics of the study cohort.

### 3.2. Association Between Clinical Variables and Malaria Infection

Based on the Chi-square test results, it is evident that there are varying levels of association between different variables and malaria status as shown in Table 1. Hemoglobin levels were categorized with a cutoff value of Hb < 11 g/dL and Hb > 11 g/dL to be anemic and non-anemic, respectively. In addition, children who had a fever were categorized as YES and those without fever as NO. When examining the relationship between fever and malaria status, the *p*-value of less than 0.001 indicated a strong and significant association between the two variables. Additionally, the hemoglobin level and malaria status showed a borderline significance with a *p*-value of 0.053.

### 3.3. Comparing Micronutrient Biomarkers Levels Between Malaria Infected and Healthy Children

Results, as shown in Table 2, indicated a significant difference in ferritin levels between malaria-infected and healthy children (t = −3.182, *p* = 0.002). Specifically, malaria-infected children had a higher mean ferritin concentration (M = 23.53 µg/L) compared to those that were healthy (M = 19.07 µg/L), with a mean difference of 4.47, and a 95% confidence interval ranging from −7.27 to −1.67. Additionally, a significant difference was also observed in soluble transferrin receptor (sTfR) levels between the two groups (t = −2.003, *p* = 0.049), with malaria-infected children having a higher mean sTfR concentration level (M = 3.74 mg/L) compared to the healthy children (M = 3.08 mg/L), resulting in a mean difference of 0.66mg/L. However, the mean concentration levels of RBP4 and Tg were high in healthy children (RBP4 M = 0.83 µmol/L, Tg M =20.91 µg/L) compared to those that were infected with malaria (RBP4 M = 0.82 µmol/L, Tg M = 19.98 µg/L), but their difference was not statistically significant.

### 3.4. Categorization of Micronutrient Biomarkers into Low and Normal Levels Amongst Malaria Infected Children

**Figure 3:**
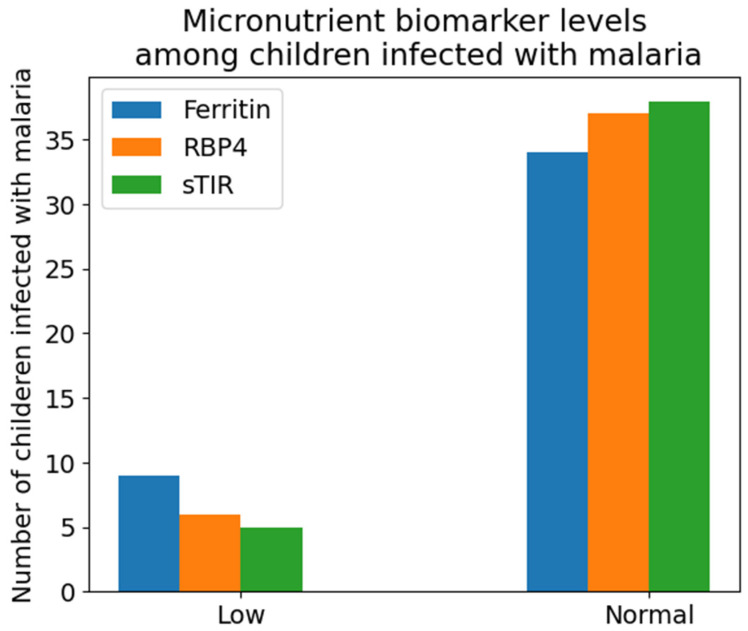
Categorization of micronutrient biomarkers into low or levels in amongst children infected with malaria.

The micronutrient biomarkers levels of children infected with malaria were categorized as low or normal as shown in Figure 3. Ferritin, RBP4, and sTfR were categorized as; ferritin < 15 µg/L to be iron storage deficiency [25], RBP4 < 0.7 µmol/L [26], functional vitamin A deficiency and sTfR > 8.3 mg/L [27] as functional iron deficiency. From Figure 3, results showed that nine (20.9%) of the children infected with malaria had iron storage deficiency, six (14%) had functional vitamin A deficiency and five (11.6%) had functional iron deficiency.

### 3.5. Logistic Regressions Analysis of the Effects of the Study Biomarkers on Malaria Infection Outcome

Table 3 shows that males were 12.3% more likely to experience the outcome compared to females, when other variables were held constant. However, *p*-value was much higher than 0.05, meaning that the effect of sex was not statistically significant. Based on Beta coefficient, the positive beta coefficient suggested that older children were slightly more likely to experience malaria infection. In addition, based on *p*-value of 0.022, age was significantly associated with the parasite load (i.e., malaria positive/negative), and hence it can be considered as a significant risk factor for the parasite load.

A negative beta coefficient of −0.2672 functional iron deficiency indicated that as hemoglobin increases, the odds of having malaria decreases, reinforcing the idea that higher hemoglobin concentration could be a sign of being healthy. However, *p*-value is much higher than 0.05, meaning that the effect of hemoglobin is not statistically significant.

Based on an odds ratio, for each unit increase in ferritin levels, the odds of having a malaria infection increased by 2%. However, a *p*-value much higher than 0.05 indicated that the effect of ferritin was not statistically significant.

### 3.6. Correlation Heatmap of the Study Micronutrient Biomarkers

**Figure 4:**
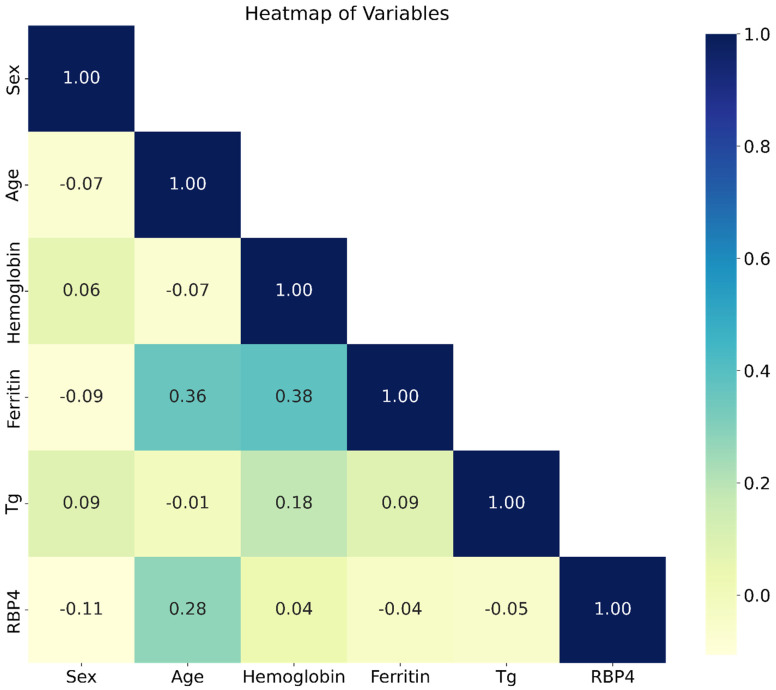
Correlation Heatmap of the study micronutrients and variables. Python was used to generate a HeatMap to show the correlation between the study variables and one another.

A heatmap was used to visualize the correlation between the various study micronutrients correlated with each other as carried out in the present study. Ferritin and Age had a moderate positive correlation (r = 0.36), suggesting that as age increased, ferritin levels tend to increase (Figure 4). Ferritin and Hemoglobin also showed a moderate positive correlation (r = 0.38), implying that higher hemoglobin levels were associated with higher ferritin levels. Most other relationships between variables showed weak correlations (values closer to 0), indicating little or no strong association.

## 4. Discussion

Malaria infection can affect the levels of micronutrients in the serum, and the deficiency of these micronutrients in the serum can increase the risk of having a malaria infection [28]. This study was carried out in the Buea Health District to ascertain the situation in this locality, and it was observed that serum micronutrient levels and malaria infection affected each other.

In Table 2, the results showed that of the 37 healthy children, 16 of them had an HB < 11 g/dL meaning they were anemic. Since these children were not diagnosed with malaria, one of the possible causes of this could be their diets. This could be that some of the foods consumed by the children do not promote the production of RBCs or are poor in iron, which is needed to produce hemoglobin.

This study showed that ferritin mean concentration levels were significantly higher in children diagnosed with malaria: 23.53 µg/L (SD 7.75), compared to the healthy children, with a mean difference of 4.47 µg/L. This result was expected, as ferritin levels are affected by infections like malaria. This then means that some of the children infected with malaria could have had a high production of ferritin, which could be due to the demand for iron by the stem cells, which can be used to produce RBCs and immune cells against the infection. However, after categorizing the ferritin levels using the cutoff point, ferritin < 15 µg/L to be iron storage deficient [29], 9 of the malaria positive patients were storage iron deficient and 34 had elevated ferritin levels. The reason for the nine children having iron storage deficiency could be because most of the iron stores were used for immune cell development against the malaria parasite or they did not have adequate iron stores even before the infection. This result was similar to those from a similar study conducted by Barffour et al. [30] in Zambia on children below 5 years old, who had ferritin levels significantly higher in malaria-sick children compared to healthy children.

After categorizing using sTfR > 8.3 mg/L [31], five children that were diagnosed with malaria, were found to have elevated sTfR levels. This means that these six children had functional iron deficiency. This could be because more iron was needed to produce RBCs and immune cells against malaria parasites and because they had inadequate levels of iron, the levels of sTfR increased above normal. This finding is in line with the results from Zambia by [30], with sTfR levels significantly higher in malaria infected children compared to those that were healthy, when assessing sTfR and ferritin levels in children below 5 years old.

RBP4 mean concentration levels were high in healthy children 0.83 µmol/L (SD 0.08), compared to in children that were sick with malaria 0.82 µmol/L (SD 0.14), having a mean difference of 0.01 µmol/L. However, the difference between the two mean levels was not significant. This result was not in line with the findings of Das and colleagues [32], as they had significantly low levels of RBP in malaria-infected children compared to those that were healthy. One of the possible reasons for this could be the number of samples analyzed. However, after categorizing RBP4 levels, the cut off value RBP4 < 0.7 µmol/L [33] for functional vitamin A deficiency, six children diagnosed with malaria had functional vitamin A deficiency. This could be because the body might use more vitamin A during a malaria infection to produce immune cells against the malaria parasite.

Thyroglobulin synthesis requires iodine, and so low levels of iodine will lead to decreased production of the thyroid hormone [20]; thus, in order to maintain adequate hormone levels, the thyroid gland increases thyroglobulin production in response to low iodine levels [21]. Thyroglobulin mean concentration levels were high in healthy children, 20.91 µg/L (SD 2.49), compared to the malaria-infected children, with a mean difference of 0.91 µg/L, but it was not significant. This could be because malaria parasite infection affects the functioning of the thyroid gland, suppressing thyroid hormone production and consequently low thyroglobulin.

## 5. Conclusions

In conclusion, this study shows that malaria infection altered serum levels of micronutrient biomarkers, and subsequently micronutrients with ferritin and sTfR were significantly higher in children infected with malaria compared to those that were healthy in the Buea Health District. The main limitation of this study was the small number of samples analyzed, and therefore it is recommended that the research be repeated with a larger sample size and with others unstudied yet for malaria micronutrients. Also, in this research the different types of malaria parasite species diagnosed were not taken into consideration. Therefore, this should be considered while repeating the research. Future research could include assessing additional micronutrients or adopting a longitudinal study approach, which could provide more comprehensive insights into the relationship between malaria and micronutrient status.

## Figures and Tables

**Table 1:** Association between clinical variables and *P. falciparum* malaria infection.

		Malaria Status		
Variable	Category	Negative (%)	Positive (%)	*p*-Value
Fever	No	37 (46.3)	0 (0)	<0.001
	Yes	0 (0)	43 (53.8)	
	Total	37 (46.3)	43 (53.7)	
Hemoglobin level	Anemic	16 (20)	27 (33.7)	0.053
	Non-Anemic	21 (26.3)	16 (20)	
	Total	37 (46.3)	43 (53.7)	

**Table 2:** Comparison of micronutrient biomarkers means between malaria infected and healthy children.

Biomarker	Malaria	Mean	SD	t	*p*-Value	Mean Difference	95%CI	
							Lower	Upper
RBP4 (µmol/L)	Negative	0.83	0.08	0.51	0.673	0.01	−0.038	0.064
	Positive	0.82	0.14					
Ferritin (µg/L)	Negative	19.07	3.87	−3.182	0.002	−4.47	−7.27	−1.67
	Positive	23.53	7.75					
Tg (µg/L)	Negative	20.91	2.49	1.309	0.194	0.93	−0.49	2.35
	Positive	19.98	3.64					
sTfR (mg/L)	Negative	3.08	0.64	−2.003	0.049	−0.66	−1.32	−0.004
	Positive	3.74	1.92					

**Table 3:** Odds ratios and effects of micronutrients biomarkers on malaria infection outcome.

Variable	Odds Ratio	95% Confidence Interval	*p*-Value	Beta Coefficient	Effect
Male vs. Female	1.123	[0.465, 2.709]	0.796	0.1160	Positive
Age	1.037	[1.005, 1.069]	0.022	0.0362	Positive
Hemoglobin	0.766	[0.555, 1.057]	0.104	−0.2672	Negative
Ferritin	1.02	[0.972, 1.069]	0.422	0.0194	Positive
Tg	0.893	[0.770, 1.036]	0.136	−0.1127	Negative
RBP4	0.878	[0.495, 1.558]	0.656	−0.1302	Negative

## Data Availability

Dataset used for paper is available upon request from the corresponding author.
